# Assuring the quality of programmatic assessment: Moving beyond psychometrics

**DOI:** 10.1007/s40037-018-0485-y

**Published:** 2018-11-08

**Authors:** Sebastian Uijtdehaage, Lambert W. T. Schuwirth

**Affiliations:** 1 Graduate Programs in Health Professions Education, Uniformed Services University of the Health Sciences, Bethesda, USA; 2 Prideaux Centre for Research in Health Professions Education, College of Medicine and Public Health, Flinders University, Bedford Park, South Australia Australia

The concept of programmatic assessment in health professions education was introduced in 2005 [1] and is rapidly gaining traction. Its central tenet is appealing: assessing learners longitudinally with a variety of methods that are embedded in the educational process, and that afford both assessment *of* learning and assessment *for* learning. Programmatic assessment accommodates both low-stake and high-stake decisions and is reminiscent of what a vocal coach does: he helps to make a singer achieve his or her utmost potential (by giving frequent feedback) but eventually will make a summative decision whether the singer can join the choir or can be the soloist.

While some aspects of programmatic assessment extend existing assessment practices, others are quite new. For instance, continuous and longitudinal assessment, which is part of programmatic assessment, is not new; progress testing was introduced in the 1970s and applies the principle of repeated assessments in the knowledge domain with multiple-choice tests. Also, the combined use of multiple assessment formats is not new. In workplace-based assessment, for example, combining direct observation and 360-degree feedback, as well as the use of portfolios, have been around for much longer.

Three aspects, however, are quite unique and fundamental to programmatic assessment: (1) meaningful triangulation across instruments; (2) proportionality of decision making; and (3) diversity of quality assurance processes.

**Meaningful triangulation** means that information about a student’s strengths and weaknesses is collected across different assessment methods rather than within. Traditionally, performance on one activity is averaged with another activity because they are assessed with the same format. This practice, however, is problematic. For instance, averaging the score for knee examination with the score for stomach examination assessed in two different OSCE stations does not make much sense and is not helpful to the learner. Instead, combining assessment information from different sources (both quantitative and qualitative) and seeking additional ‘diagnostics’ if needed (the triangulation aspect) result in more meaningful conclusions. For example, poor performance on a knee examination in an OSCE is better understood in light of information gleaned from relevant parts of a multiple-choice exam.

**Proportionality** means that the stakes of an assessment decision is commensurate with the richness and trustworthiness of the information on which the decision is based. Deciding that a learner needs to review content (a low-stake decision) can be based on one or few assessments. On the other hand, deciding that a learner needs to redo a clerkship (a high-stake decision) should be based on reliable, trustworthy information gleaned from repeated assessments in a variety of formats.

The triangulation and proportionality aspects of programmatic assessment have important implications for **quality assurance** and how we collect validity evidence. After all, it is not only the quality of the individual assessment methods that is relevant, but also how they are combined and add value to the program as a whole. This requires a fundamentally different approach to validity and a ‘move into a “post-psychometric era”’ [2].

Michael Kane’s validity framework is particularly well-suited for programmatic assessment [3]. Briefly, Kane breaks down an assessment process into four sequential stages, each of which rests on a set of assumptions and inferences: (1) scoring; (2) generalization (the focus of the study in this issue by Bok et al. [4]); (3) extrapolation; and (4) implication. It is incumbent on those who make assessment decisions to build a coherent and plausible argument by prioritizing evidence for each of these inferences. Kane further describes that evidence on which the argument is based could be empirical, but could also be logical. According to Kane, the fundamental building blocks for each argument are clarity, coherence, and plausibility. We refer the reader to Cook et al. [2] for an excellent introduction to Kane’s framework and to Schuwirth & Van der Vleuten [3] for a description of Kane’s validity perspective on programmatic assessment.

In this issue, Bok and colleagues [4] take a purely psychometric and statistical approach to examining the validity of a program of assessment. Building on Kane’s framework they focus on the generalization inference of three work-based assessment methods that were administered weekly in the course of almost two years. The findings of their sophisticated analysis could certainly be brought to bear in a validity argument, but should not be seen as a final and complete argument. Assessing clinical competence typically involves the collection of information that is numerical (i. e. scores) as well as descriptive (i. e. rich narratives). Consequently, the concept of ‘generalizability’ takes on a different meaning in programmatic assessment and is maximized by repeated and deliberate sampling of numerical and descriptive assessment information by trained, credible assessors until saturation of information has been achieved [5].

The process that starts with observing a student’s performance and ends with making a summative decision about that student’s clinical competence is a long road fraught with peril. Numerous quality assurance issues must be looked at. Is the assessment program acceptable and transparent to all stakeholders, including the public? Does the committee who makes high-stake decisions have the expertise and qualifications to synthesize complex assessment information? How do they make such decisions? Is the programmatic approach feasible at all and are the costs reasonable? Does the assessment program have unforeseen consequences to learners or patients? These questions (among many others) point to one or more of the four inferences in the assessment process. Kane’s nuanced and flexible approach to validity suggests not only a wealth of research questions but does also justice to a process that is as complex and multifaceted as programmatic assessment.
